# Scope: A new service supporting family doctors dealing with psychiatric patients in the community: Current utilization and quality improvement implementation protocol in the covid era

**DOI:** 10.1192/j.eurpsy.2021.1047

**Published:** 2021-08-13

**Authors:** L. Hytman, B. Bolea-Alamañac

**Affiliations:** Psychiatry, The Women’s College Hospital Institute for Health System Solutions and Virtual Care (WIHV), Toronto, Canada

**Keywords:** Virtual Care, COVID-19, mental health

## Abstract

**Introduction:**

Seamless Care-Optimizing Patients Experience-Mental Health (SCOPE-MH) is a hub-based integrative case management and psychiatric care program supporting family physicians (FPs). SCOPE-MH provides patient resource navigation, social support, counselling, psychiatric consults, and short-term follow-up. Due to COVID-19, SCOPE-MH pivoted to serve patients completely online.

**Objectives:**

To assess current utilization and evaluate patients’ and FPs’ experiences using SCOPE-MH as an online service before and during COVID-19.

**Methods:**

This evaluation was developed under the RE-AIM framework (Reach, Adoption, Implementation and Maintenance). Two surveys, one for Patient Reported Experience Measures (PREMS), and one seeking FPs perspective on the service, will complement the evaluation.

**Results:**

Past data showed that 66.4% of referrals to SCOPE-MH were women (ages 14-97), and 33.6% were men (ages 14-91). The most common diagnoses were anxiety and depression, followed by adjustment reaction and PTSD. 72% of referred patients had more than one psychiatric diagnosis. 35.4% of the referrals were resource navigation and brief coordination of care. 39.2% required long term involvement. The main recommendations provided were counselling resources in the community and referral to local community mental health teams. Data on patient and FP experiences using SCOPE-MH, and perspectives on unique needs for psychiatric care in COVID-19, is still being collected. Surveys will be sent within 6 months.

**Conclusions:**

SCOPE-MH is an effective model to support FP’s addressing patients’ psychiatric needs. The information obtained from the evaluation will be used to modify the online service to address unmet needs during COVID-19 and optimize current resources to serve more patients.

